# Lessons from implementing mass drug administration for soil transmitted helminths among pre-school aged children during school based deworming program at the Kenyan coast

**DOI:** 10.1186/s12889-017-4481-7

**Published:** 2017-06-14

**Authors:** Rosemary M. Musuva, Elizabeth Matey, Janet Masaku, Gladys Odhiambo, Faith Mwende, Isaac Thuita, Jimmy Kihara, Doris Njomo

**Affiliations:** 1 Center for Global Health Research (CGHR), Kenya Medical Research Institute, P. O. Box 1578-40100, Kisumu, Kenya; 2 Eastern and Southern Africa Centre of International Parasite Control (ESACIPAC), Kenya Medical Research Institute, P. O. Box 54840 – 00200, Nairobi, Kenya; 3 Center for Microbiology Research, Kenya Medical Research Institute, P. O. Box 54840 – 00200, Nairobi, Kenya; 4 Ministry of Education, Directorate of Basic Education, Early Childhood Education Section, P. O. Box- 30040-00100, Nairobi, Kenya; 5 Ministry of Health, Division of Vector Borne Diseases, P. O. Box-20750-00202, Nairobi, Kenya

**Keywords:** Soil transmitted helminthes, Mass drug administration, Pre-school teachers, School based deworming

## Abstract

**Background:**

The 2012 London declaration which committed to “sustaining, expanding and extending drug access programmes to ensure the necessary supply of drugs and other interventions to help control soil-transmitted helminths (STH) by 2020” has seen many countries in Africa roll out mass drug administration (MDA) especially among school age children. In Kenya, however, during the National school-based deworming exercise, pre-school aged children (PSAC) have to access treatment at primary schools as the pre-school teachers are not trained to carry out deworming. With studies being conducted on the effectiveness of MDAs, the experiences of key education stakeholders which could improve the programme by giving best practices, and challenges experienced have not been documented.

**Methods:**

This was a cross-sectional qualitative study using Focus group discussions (FGDs) and Key informant interviews (KIIs). It was conducted in 4 sub-counties with high STH prevalence at the Kenyan coast (Matuga, Malindi, Lunga Lunga and Msambweni) to understand best practices for implementing MDA among PSAC.FGDs categorized by gender were conducted among local community members, whereas KIIs involved pre-school teachers, primary school teachers, community health extension workers (CHEWs) and opinion leaders. Participants were purposefully selected with the saturation model determining the number of interviews and focus groups. Voice data collected was transcribed verbatim then coded and analyzed using ATLAS.Ti version 6.

**Results:**

Majority of the primary school teachers and CHEWs reported that they were satisfied with the method of mobilization used and the training tools. This was however not echoed by the pre-school teachers, parents and chiefs who complained of being left out of the process. Best practices mentioned included timely drug delivery, support from pre-school teachers, and management of side effects. Overcrowding during the drug administration day, complexity of the forms (for instance the ‘S form’) and long distance between schools were mentioned as challenges.

**Conclusion:**

There is need to utilize better sensitization methods to include the local administration as well as the parents for better uptake of the drugs. Extending deworming training to pre-school teachers will enhance the national deworming programme.

**Electronic supplementary material:**

The online version of this article (doi:10.1186/s12889-017-4481-7) contains supplementary material, which is available to authorized users.

## Background

The current World Health Organization (WHO) recommended strategy for control of soil-transmitted helminths (STH) at the community level involves targeted distribution of albendazole (ALB) or mebendazole (MLB) based on the prevalence of infection in school-aged children (SAC) [1–3]. The strategy also recommends treatment of preschool aged children (PSAC), women of childbearing age and adults at high risk in certain occupations (e.g. tea-pickers and miners) [4]. Based on these recommendations, several STH-endemic countries have rolled out MDA programs that have targeted mainly SAC, leaving out PSAC and adults at high risk. SAC are considered ideal subjects for surveys and interventions because schools are easily accessible and it is cost-effective. SAC bear the highest burden of infections, prevalence and intensity levels. The SAC are a representative of the community [5] and therefore, can be used to make intervention decisions. However, it is noteworthy that PSAC comprise between 10% and 20% of the 3.5 billion people living in STH-endemic areas [6], and there is an increasing appreciation that PSAC may be an important reservoir of STH infection. In its strategic plan on “Eliminating soil-transmitted helminths as a public health problem in children”, the WHO aims to increase global PSAC deworming coverage to 75% by 2020 [7].

Although MDA programs have been shown to be largely effective against helminths, transmission has not been effectively interrupted by MDAs especially for high risk locations [8, 9]. Whereas MDA programs have reduced prevalence to appreciable levels in some areas, prevalence has remained high in other areas, or the reduction in prevalence has been less than what was anticipated in other areas (SCORE program, western Kenya, unpublished data) and that in some, the reduction in prevalence leveled off at some point or increased [10–13]. The sustained prevalence in some of these areas might be attributable to among other factors, the efficacy of the drugs, the influence of local environmental and behavioral factors on individual risk for primary infection and/or reinfection, but also on the presence of reservoirs of infection including PSAC that are often left out during MDA. Furthermore, other program-specific factors might also contribute to the performance of a control program, and a collective understanding of all these factors is critical in improving ongoing control programs and designing new ones.

In Kenya, the National School-Based Deworming Programme (NSBDP) was launched in 2009 (with albendazole) and 2012 (with PZQ) administered in prioritized areas, and by 2013 over 3.6 million SAC had been treated [14]. The stand alone pre-schools receive treatment at the nearby primary schools, some of which are over 5 km away. Other deworming interventions are carried out through Ministry of Health programs such as *Malezi bora* (proper nurturing) provided at ante-natal clinics. The Kenya NSBDP has been running for several years, it is important to assess the levels of successes and challenges experienced to improve intervention decisions. Moreover, while there is a lot of data on MDA activities among SAC, there is much less information on factors affecting treatment among PSAC. Some of the lessons learnt from MDA among SAC which would improve interventions include the use of national health weeks [15], child health days [16], government ownership, utility of a multi-sectoral coordination committee, support by development partners and close communication and collaboration among different state government bodies, in particular the health and education sectors [17]. Context-relevant community awareness campaigns that built local ownership of the programme have also been reported to result into high levels of acceptability of the deworming programme by the community [17].

This study sought to document the experiences of key stakeholders on best practices and their challenges from STH deworming activities conducted in 4 sub-counties at the Kenyan coast in a bid to improve deworming treatment in preschool aged children. This cross-sectional qualitative study was part of a larger study that evaluated different drug delivery approaches for treatment of STH in PSAC. With an average 55.6% of rural pupils in Kenya travelling for more than 5 km to reach the nearest school, there is a need therefore to develop and implement an alternative drug delivery method in order to help maximize treatment coverage for this vulnerable age group. The lager study therefore sought to test two methods (1) engaging and facilitating the CHEWs to visit the standalone ECD Centres to treat the children and (2) having the ECD Centre teachers and parents take the children to the nearby primary school for treatment as currently proposed by the programme. The two methods were used to determine the cost effectiveness of both methods, compare treatment coverage between the two methods and to develop an alternative drug delivery method for the PSASC. Findings from such surveys are important in understanding how the economic and social-behavioral factors influence control programs in order for countries to adequately adapt the recommended WHO STH control strategy to local situations.

## Methods

### Study area

This study was conducted in 4 sub- counties at the Kenyan coast – Matuga, Msambweni, Lunga Lunga and Malindi. These counties are populated primarily by members of the *Miji-Kenda* community and were selected on the basis of high prevalence for STH infections. The results of a survey conducted prior to deworming in 2012 showed that the prevalence of STH in pre-schools in Matuga and Msambweni sub-counties in Kwale County was 27.8 and 66.7% respectively while that of Malindi sub-county in Kilifi County was 44.5% [18]. Though there are many development initiatives in these areas, poverty is still a major challenge. As in many other low and middle income countries, health care delivery in Kenya is based on the Primary Health Care concept [19]. Treatment of STH infection in health facilities is mostly based on identification of STH ova using the direct smear method or on signs and symptoms (presumptive treatment) and an out-of-pocket system of payment (the patient is required to make full payment for consultation before treatment is provided). Most essential drugs are kept in the health facilities for purchase, but patients have to obtain other drugs from private chemists/pharmacies [20].

### Study design

A cross-sectional design adopted. It was conducted in all stand-alone (not sharing the school compound with a primary school) pre-schools in the 4 sub-counties. By use of Geographical Information Systems (GIS), Quantum [21], all preschools that were stand alone and 2 or more kilometers away from a primary school in the 4 sub-counties were mapped out and considered for the study. Fifty percent of the pre-schools in each sub-county were assigned for treatment by the primary school teachers of the mother/nearby schools while the remaining half were assigned for treatment by the CHEWs.

Five focus group discussions (FGDs) stratified by gender were conducted among local community members in each sub-county. The FGDs were conducted in *Kiswahili*, which is the language commonly used by the Coastal people. Community health workers (CHWs), familiar with the villages, helped to mobilize participants for the study who were purposefully selected. The study participants, whether male or female had to be above 18 years old. Further screening was done on site to make sure that participants met the inclusion criteria before seeking informed consent and that they were a true representation of the various villages in the sub counties. The research team, together with other experts in the field of qualitative research, designed a semi-structured FGD guide (Additional file 1) that provided a general overview of the topics. Discussions were steered by a moderator who was part of the field team and had undergone training before the data collection exercise. The moderators were free to probe for additional information depending on the participant’s responses and areas that the moderator felt needed more information. In addition to FGDs, Key informant interviews (KII) were conducted with pre-school and primary school teachers, CHEWs, public health officers and opinion leaders. We used the random purposeful method to select primary and pre-school teachers in the various sub-counties. Purposive sampling was employed for the CHEWs, public health officials and opinion leaders. This category of people had been involved in the study either through drug administration or sensitization process and their feedback was critical for this study. The discussion guides covered: teacher, parent and CHEW perception of the deworming programme, sensitization methods employed, challenges in drug distribution, what worked and proposed way forward (Additional file 2). FGDs were conducted in class rooms or community halls in the respective sub counties. KII were conducted at the participant’s convenient location. These included: chief’s office, public health offices in the hospital and secluded offices in the school compound. All voice data from the FGDs and KIIs were tape recorded and later transcribed. The information was used to create a detailed reconstruction of experiences of key stakeholders on what works, and their challenges pertaining to control of STH infection among PSAC during school-based deworming program.

### Data analyses

Transcripts were first created in *Kiswahili* the local language, translated into English, and back-translated into *Kiswahili* to ensure that the English and local language versions carried the same meanings. The coding structure evolved inductively with the codes from the narrative data of earlier interviews informing subsequent coding of the following interviews supplemented with field notes from the interviewer and note taker. A coding frame was developed through open coding, a word-by-word analysis used to identify, name, and categorize explanations and descriptions of the day-to-day reality of participants as related to their perspectives of treatment of the pre-school aged children. Consensus on the coding frame was obtained through discussions between the two research assistants who had also participated in collection of the data. Each individual transcript was then examined to identify texts relevant to the coding frame. Quotes were later retrieved from the output monitor and arranged according to themes. Our data was validated by the triangulation in methodology, verification of transcripts with the audio files and discussions on the coding systems until agreements were reached.

## Results

### Socio-demographic characteristics of the study population

A total of 203 and 154 individuals participated in 20 FGDs and KIIs respectively, in the 4 sub-counties. Key informant interviews (KII) were conducted with 41 pre-school and 38 primary school teachers, 15 CHEWs, 20 public health officers and 40 opinion leaders.

Tables 1 and 2 show the socio-demographic characteristics of the participants in the study.

**Table 1:** Socio-demographic characteristics of the study participants in the FGDs

Description	Frequency (*n* = 203)	Percentage (%)
Gender		
Male	89	43.8
Female	100	49.3
Missing	14	6.9
Age in years		
15–19	2	1.0
20–24	36	17.7
25–29	30	14.8
30–34	39	19.2
35–39	35	17.2
40–44	18	8.9
45–49	18	8.9
≥ 50	24	11.8
Missing	1	0.5
Educational level		
Primary education^a^	105	51.7
Secondary education^a^	14	6.9
None	47	23.2
Missing	37	18.2
Religion		
Christianity	89	43.8
Islam	107	52.7
None	4	2.0
Missing	3	1.5
Occupation		
Business	40	19.7
Farming	111	54.7
Fishing/Fish monger	3	1.5
Housewife	27	13.3
Casual laborer	9	4.4
Religious leader (Pastor or Imam)	3	1.5
Community health volunteer	1	0.5
Skilled laborer	2	1.0
Village elder	1	0.5
Teacher	3	1.5
Missing	3	1.5

^a^Includes people who received some education but may not have completed this level

**Table 2:** Socio-demographic characteristics of the study participants in the KIIs

Description	Frequency (*n* = 154)	Percentage (%)
Gender		
Male	95	61.7
Female	59	38.3
Age in years		
20–24	8	5.2
25–29	21	13.6
30–34	25	16.2
35–39	15	9.7
40–44	20	12.9
45–49	24	15.6
≥ 50	41	26.6
Marital status		
Single	26	16.9
Married	126	81.8
Divorced	2	1.3
Religion		
Christianity	79	51.3
Islam	72	46.8
Missing	3	1.9
Occupation		
Chief/Assistant Chief	14	9.1
Business	5	3.2
Farmer	2	1.3
CHEW	14	9.1
Religious leader (Pastor or Imam)	5	3.2
Public health officer	20	13.0
School chairman	3	1.9
Village elder	9	5.8
Primary school teacher	38	24.8
Preschool teacher	41	26.6
Youth leader	2	1.3

## Perception of the school based deworming Programme

Participants were receptive of the drug distribution exercise. Teachers mentioned marked improvement in the health of the children after treatment. *“..Some children in the school, when you looked at them, their health had deteriorated but now they seem to be doing better...those drugs are helping us.”* Teacher (Malindi)

Majority of the participants observed that the drug distribution in the preschools went well. However, they strongly suggested that future exercises should utilize the pre-school teachers instead of the CHEWs *“Administration of drugs should be done in the preschools and Community health workers are ok but the preschool teachers should be taken for seminars to be trained because the CHEWs are strangers to the children but teachers can do it easily. The children identify well with the teachers so the CHEWS should supervise but teachers to administer”.* Opinion leader (Matuga).

*“I think the best is the teacher because he/she is familiar with the children, they know their weaknesses and their characters at large because he/she is with them most of the time but if we say for instance the CHEW when they get there how will they know that a particular child has a condition, epilepsy for instance, they will just give them the drugs right? so in the end the CHEW will still need the preschool teacher for things to go well.”* Primary school teacher (Matuga).

## Sensitization strategies

### Teacher trainings before drug delivery

Findings from the key informant interviews with pre-school and primary school teachers revealed that multi-sectoral involvement was important and they mentioned this as one of the factors that encouraged them to participate in the deworming exercise. They indicated that the presence of the District Officer (DO), Area Education Officer (AEO), Ministry of health officials and head teacher was proof that the programme had been approved. A teacher from Matuga said, *“The head teacher is usually the first to be informed by the authorities maybe by the DO’s or the AEO’s office. After that, the head teacher then informs me and we attend the seminar. When you go there and meet all those seniors, some from the Ministry of Education, others from the Ministry of Health, you relax. You are not worried when you go back and treat the children”.*

### Community sensitization

The FGDs with community members revealed that most of the parents had not been informed about treatment by the treatment day. *“We want to know when the treatment will happen. Anything that happens suddenly scares people. Many children did not take the drugs because the exercise was so sudden and people also did not consider themselves sick. But if they had been involved before, they would know they have a certain sickness and that children would be given drugs on a particular day”* Female (Msambweni).

Some parents also faulted the method of sensitization used, which they said was not effective and suggested the use of village chairmen “*That sensitization method did not work. In order for them to understand, there has to be coordination. For it to succeed, you have to go through the provincial administration then from there, the village elders who will talk to the village chairmen.* Male (Msambweni)

Other participants reported that posters were only placed in hospitals and went on to argue that only women got that information since they are the only ones who go there.

### Pre-school teacher sensitization

Our study revealed that the pre-school teachers were not included in the training exercise. In this case, some were not aware of the treatment day and did not show up in school. *“In another school that we went to, the teacher had not reported to school that day, it was a pre-school. He/she had gone to attend a certain meeting so since the teacher was absent, children too decided not to report to school”* CHEW (Matuga).

## Challenges in drug distribution

### Overcrowding in schools during treatment day

Majority of the teachers complained of overcrowding in the primary schools especially with children from the pre-schools. *“You know this school has many feeder pre-schools. So we ended up having children from four schools coming to get treatment. You could see the teachers were overwhelmed. Some children were crying, some were tired of standing. It would have been better if they were treated from their own school “*Primary school teacher (Lunga Lunga).

### Long distances to mother schools and time allocated for treatment

Due to the distance to primary schools, some pre-school teachers had to treat their pupils, yet they had not received training on treatment.

*“Some children can't handle the distance to far places…it becomes hard, also the parent is never sure if the child has eaten anything or not yet they are expected to take the drugs, so that can affect them because they need energy to make the journey. if the child has to go to a school where there is a main road then it becomes a problem, because the teacher has to help them cross the road and the children are so many for that one teacher to help them all.”* Opinion leader (Matuga).

Even though most schools conducted treatment, some pre-schools missed out on treatment completely. *“I am aware of some preschools that missed out on the treatment completely…..I think it is because of the mushrooming of some of these schools. I think the people giving the drugs did not know the schools existed”*, stated a pre-school teacher from Msambweni

Parents felt that the time allocated for the exercise was not enough especially for the non- enrolled who need to travel long distances to seek treatment in the schools.

*“It is too short. The parents did not have enough time to prepare the children to go. For example the mother gets information today about the treatment and she had some business in the market. When she goes there tomorrow she is told the exercise ended yesterday.”* Opinion leader (Malindi).

### Fear of the drug

A primary school teacher from Msambweni expressed his concerns “*… at the beginning they were afraid of coming forth because them they knew we were administering praziquantel and from experience they know praziquantel is bitter in taste so however much we tried telling them that it's not praziquantel they would still decline because they were convinced its praziquantel so they would hide so that was a problem.”*

### Filling in of record keeping forms

There was an emphasis on the volume of workload related to the treatment exercise. A health teacher from Msambweni sub-county said, *“It’s a lot of work. The forms are many and very detailed. You have to fill, then go back and do some math, write those down”*.

*“It was difficult and time consuming… cumbersome and involving.”* health teacher (Matuga).

## Best practices

### Drug distribution in the schools

The teachers reported that getting the drugs at the end of the training session which was a week before the treatment date worked for them as opposed to collecting them from a central location on the material day of treatment. A teacher from Lunga Lunga said, *“We were given the drugs on time, in fact that very day of the meeting. It was very early, like one week before the treatment date so there was time to distribute to the other teachers”*.

These sentiments were echoed by a CHEW from Matuga “*The drugs were received earlier before the day of treatment which was nice as it could at least take care of any inconveniences in transporting and availing the drugs and also treating at the right time …”*

### Close supervision by health workers

Although side effects were mentioned as a challenge during the drug distribution exercise, having a public health officer within reach to assist when complications arose was cited as a boost in the exercise.

*“… the advantage we had is the fact that the health worker was just from within so anytime we had a problem we would communicate with him/her and everything was solved.”* health teacher (Matuga).

## Proposed way forward

### Feeding before taking the deworming drugs

It was suggested that the treatment exercise be timed to coincide with the harvest season when there was plenty of food**.** A health teacher from Lunga Lunga said*, “During the training we were told that the children need to eat something. So we made that announcement. We told them to eat before coming to school. But when you look around, it was a bad time. There was no food in the farms. We knew that would be a problem…a feeding programme would help”*.

### Frequency of drug distribution

Parents felt strongly that the drug distribution should take place more often than the once in a year schedule. This was pegged to the frequent reinfection they noticed.

*“… later we observed in some kids when they walked you could see the worms coming out from the rear behind, those that were infected by worms so I would say the exercise was effective and if they dewormed after every short while it will be so good because some worms reappear again after the 6 months.”* Male (Lunga Lunga).

*“It is important to get the drugs because that disease here does not go away, one just gets better. You feel better and within no time, you get the same problems. You take the drugs, but after some time the drugs are not effective anymore, the worms are back.”* Female (Matuga).

### Community sensitization strategies

Participants indicated that the sensitization methods employed by most schools had shortcomings. The children either forgot to give the information to parents or did not understand what the MDA was about. Suggestions given include; road shows, radio, village chairmen and chiefs baraza.

*“…Yes like we have the Kaya FM, Bahari FM which is really listened to. The local administration the chief and so forth. The local leaders, the church and the mosque can… if the community is given a message through the mosque it would be heard easily. The mosque and the church leaders it would be heard very easily.”* CHEW (Matuga).

An opinion Leader from Matuga had a different view, *“Community mobilization should be done through the chief’s baraza’s because through this many people can be reached and will be aware of the programs as they are being carried out. Parents should also be involved greatly by letting them know that they can take their non-enrolled children to the ECD centers to get treatment.”*

## Discussion

The objective of this study was to understand the experiences and challenges of key stakeholders from STH deworming activities in 4 sub- counties at the Kenyan Coast in order to improve the programme. The school-based deworming, in which the point of care for children is the school and trained school teachers undertake the role of drug distribution, with critical oversight by health care staff, is recommended in order to cost-effectively and efficiently reach large numbers of children [4]. However, individual countries need to adapt the recommended WHO STH control strategy for local situations and to allow for comparisons in subsequent monitoring and evaluation. In appreciation of this, the current study was premised on the fact that to improve ongoing STH deworming programme and design new optimal treatment strategies, incorporation of lessons from the ongoing programme and understanding of program-specific factors and challenges is essential.

### Perception of school based deworming programme in the preschools

The National School-Based Deworming Programme in Kenya was launched in 2009 [22] and over six million children received treatment by the year 2015. This great success is largely attributed the use of primary school teachers in administering drugs [14]. The gap realized in this study with pre-school teachers involvement in the training and treatment exercise is evident. Parents support the idea of children being treated in the pre-school centers to avoid trekking long distances and road hazards. This study reveals that the use of CHEWs is accepted by the community since they are health professionals, although it was also clear that CHEWs relied heavily on the pre-school teachers to identify children who did not qualify for treatment (due to age or illness) and also convince them to take the drugs because the children see the teacher as a familiar face and are more likely to trust them as opposed to a stranger.

### Sensitization

The lack of awareness on the treatment exercise by some parents despite the sensitization efforts was noteworthy. Although deworming activities in the Kenya NSBDP are conducted within deworming days set aside at the District level, creation of Child Health Days (CHD) or National Health Days (NHD) which are formally *gazetted* and clearly marked calendar activities within which deworming can be incorporated may have the added benefit of increasing awareness and improving sensitization of parents on the deworming activities. This will not only elevate the deworming activity but also give it prominence. Embedding deworming programme within CHD and NHD has been shown to work in other settings [15, 16], it will also aid in achieving global PSAC coverage targets. However, it is also acknowledged that CHD or NHD encompass provision of lots of several other health interventions, and therefore careful planning is required so as not to integrate too many interventions and compromise the planned deworming activities.

### Challenges in the deworming exercise

Majority of the parents in the present study expressed concern over their children traveling long distances to get treatment in the nearest primary school which led to some children missing school and thus deworming and rather preferred that the children be treated in their schools instead. This suggests the need for careful planning in areas where primary schools are located far away from preschools, and tailor-made solutions for each unique setting need to be utilized to enhance compliance and coverage. As a first step, mapping out of primary schools and pre-schools will help in informing decisions regarding proximity of PSACs in accessing treatment.

The fear of side effects and the bitter taste of the drug was reported as a challenge in our study. These findings are consistent with a study in Uganda [23, 24] where parents expressed their concerns over the side effects and had advised their children not to take the drugs in the coming year. However, we note that side effects are usually associated with praziquantel (the drug of choice for schistosomiasis) and not albendazole that is administered to PSAC through the NSBDP. Considering the current practice where both drugs co-administered in deworming programs, it is likely that the fears by parents may have been based on their experiences with administration of praziquantel, indicating a need for additional sensitization efforts prior to deworming. Indeed, the health belief model [25] explains that an individual’s sense of susceptibility and severity of a particular disease improves the likelihood of taking the recommended preventive health action. The community sensitization gap realized in this study during the drug distribution exercise can contribute to sub-optimal uptake of the drugs as well as the fear of the drugs as reported in another study [26]. Proposed additional methods of sensitization include: use of local radio stations, door to door campaigns, parents meetings, chiefs meetings, *barazas*, radio announcements as well as the use of village chairmen/elders.

### Successes/best practices

Intensive monitoring and evaluation have been used and inferred in other studies as a major contributing factor to the success of MDA programmes [27–29]. In our study, teachers had easy access to Ministry of Health officials whom they would reach out to in the case of any eventuality in the schools. Findings from the present study certainly contribute in identifying gaps and strengthening them to enhance deworming activities. Unlike several other NTD control programmes that cited unavailability of drugs as a major challenge [24, 30, 31], the health teachers in our study reported to have received the drugs in good time and in addition, had enough left over for non-enrolled school age children in the community. Training programme for the health teachers was described as rigorous and helpful in dealing with side effects and various eventualities on the treatment day.

## Conclusion and recommendations

In conclusion, our results show that MDA in pre-schools by CHEWs works well. CHEWs relied on the pre-school teacher to talk to the children and convince them to take the drugs as well as inform them which children were sick or had conditions that were supposed to be included in the treatment exercise, indicating the important role played by the teachers. Major concerns were also raised about children traveling long distances to get treatment in mother schools. Pre-school teachers need to be trained on drug administration since they are in a better position to monitor the children’s progress. There is need to have a mapping exercise to ensure all schools are included in the training and drug distribution exercise. Our study showed that more than half of the pre-school teachers opted to treat the children instead of having them walk long distances to neighboring primary schools for treatment, suggesting that pre-school teachers therefore need to be included in the training programme. This will not only equip them with basic knowledge on helminth infections, but it will also, place them in a position to knowledgeably execute the drug administration exercise and better prepare them to deal with side effects. Such an initiative would also take care of the overcrowding experienced in some primary schools during treatment days.

## Additional files

Additional file 1: Focus Group Discussion for Parents of ECD Children on knowledge on Intestinal Parasites and the National School-based deworming Programme knowledge and awareness Survey (DOCX 21 kb).

Additional file 2: In-depth Interview for ECD Center Teachers on their knowledge and perceptions of Intestinal Parasites and the National School-Based Deworming Programme (DOCX 34 kb).
