# Supplementary material for: Lessons from implementing mass drug administration for soil transmitted helminths among pre-school aged children during school based deworming program at the Kenyan coast
Source: BMC Public Health. 2017 Jun 14;17:575. doi: 10.1186/s12889-017-4481-7 (PMC5471907; doi:10.1186/s12889-017-4481-7)
Supplement: Supplementary file 2 — In-depth Interview for ECD Center Teachers on their knowledge and perceptions of Intestinal Parasites and the National School-Based Deworming Programme (DOCX 34 kb). [file 12889_2017_4481_MOESM2_ESM.docx]

**Additional file 2:**

**In-depth Interview for ECD Center Teachers on their knowledge and perceptions of Intestinal Parasites and the National School-Based Deworming Programme**

ID_______________________________________

Time_____________________________________

Date______________________________________

District _____________________________________

Division_____________________________________

Educational Zone **_______________________________**

| Instructions:  This form should be used for in-depth interviews with the ECD Center Teachers.  If the participants refuse to answer a question, circle the number of the question and do not mark any answers for that question.  After obtaining informed consent, read the following instructions to the participants: |
| --- |
| **“I am going to ask you questions about the National school based deworming program, so as to collect information about your knowledge of Bilharzia and soil transmitted helminthes and opinion of the National School-based Deworming Programme, your community members preferences for being reaching their ECD Children during the campaign, barriers affecting the programme and existing opportunities and outlets that could be used to reach the ECD children for treatment. Please answer the questions as honestly as you can. Your information which I will tape record will be kept private and this form will not have your name anywhere. All the information will be kept confidential until the conclusion of the study when it will be destroyed. If you have any questions or do not understand what I am asking you at any time, please ask for clarification. Some questions may prove embarrassing to you.**  **Please remember that you do not have to answer any questions that you do not want to answer and you may discontinue the discussion at any time. Do you have any questions before we begin?”** |

**Socio-Demographic Characteristics**

1. Sex (Tick)

Male (1)

Female (2)

2. Year of birth _____________________.

3. Age in Years _____________________

4. Marital Status (Tick)

Single (1)

Currently Married (2)

Type of Marriage Polygamous (1) Monogamous ( 2 )

Divorced (3)

Widow/ widower (4)

Others (Specify)___________________________

5. Religion (Tick)

Christian (1)

Islam (2)

Non-practicing (3)

Others, specify (4)__________________________

**Actual Interview (Observe - record non-verbal communication)**

**Intestinal Parasites knowledge and awareness Survey**

1.What are the common diseases in your village? Are intestinal worm infections among the common diseases in your region?

Probe for:

- Types of worms (round worms, tapeworms, bilharzia)
- Local name.

2. Where did you first learn about intestinal worms?

Probe for:

- Newspapers and magazines
- Mass media: Radio; TV; Billboards
- Brochures, posters and other printed materials
- Professionals: Health workers; Teachers
- Family, friends, neighbours and colleagues
- Leaders: At *barazas*; Religious leaders
- Other (please specify):

3. What are the signs and symptoms of intestinal worms?

Probe for:

- Symptoms associated with different types of intestinal worms

4. What would you say about diagnosis of intestinal worms?

Probe for:

- Diagnostic methods
- Acceptability
- Accessibility
- Affordability- cost
- Effectiveness

5. How does a person get intestinal worms?

Probe for:

- Life cycle
- Perceived Cause
- How it spreads

6. Who are most at risk of getting intestinal worms?

Probe for;

- Age group
- Gender
- Risky behaviors
- Occupation hazards
- What are the reasons which make you think so?

7. How can intestinal worms be avoided?

Probe for:

- Preventive and control measures
- Current interventions by community, government, others.
- What is perceived to have worked in the past and the reasons for such perceptions
- What is perceived to have failed and the reasons for such perceptions
- Acceptability
- Accessibility / Reach (define)
- Affordability

8. Are intestinal worm infections curable?

Probe for:

- Available drugs?(ALB, MBZ, PZQ)
- Cost of the drugs, Exact cost figures (perceived or real)
- How perceived (or real) costs may influence care seeking
- Herbal remedies
- Home rest without medicine
- Praying
- Any other method.
- Effectiveness of methods stated reasons for this perception

9. In your opinion, how serious a disease are intestinal parasites? Probe for:

- How serious a problem do you think intestinal parasites are in this region?
- What prompts you to think this way/ please elaborate your answer?

10. Earlier at the beginning of our discussion you listed some of your sources of information about intestinal parasites. Do you feel well informed about intestinal parasites?Probe for

- What are your other sources of information?
- How adequate do you think these sources are?
- If you could get more information about intestinal parasites, what additional information would you wish to get?
- Preferred source of information.
- Most accessible source. What makes you say this source is more accessible?

**Intestinal parasites attitudes**

11. Do you think you or the children at your ECD Center can get intestinal parasites? Probe for:

- What makes you feel you are either at risk, or not at risk of getting intestinal worms?

12. What would be your reaction if you found out that you or one of your ECD children has intestinal parasites?Probe for:

- reasons for different reactions (fear, surprise, embarrassment etc.)

13. Who would you talk to about your illness if you had intestinal parasites? Probe for:

- - - - Doctor or other medical worker
- Spouse
  - - - Parent
- Child(ren)
- Other family member
- Close friend
- No one
- Other:

Probe for:

- - - What would prompt you to talk about your illness?
    - What would make you choose a specific person to talk to about your illness?

14. Do you know of children who have/had intestinal parasites?Probe for:

- What makes you say they have intestinal worms

15. In your community, how is a child who has intestinal parasites usually regarded/treated?

**Health- seeking practices**

16. Would you go or advise a parent to take their ECD child to the health facility if you suspect you/they have intestinal parasites?Probe for:

- Place of treatment
- People to seek treatment at the places you have mentioned?
- Are certain care options more likely to be used than others?
- Under what circumstances does this happen?
- Self-medication

17. If you orone of your ECD children had symptoms of intestinal parasites, at what point should you/they go to the health facility?Probe for:

- Low
- Moderate
- Severe

18. If you/they would not go to the health facility, what is the reason? Probe for:

- Not sure where to go
- Cost
- Difficulties with transportation/distance to clinic
- Do not trust medical workers
- Do not like attitude of medical workers
- Cannot leave work (overlapping work hours with medical facility working hours)
- Do not want to find out that something is really wrong
- Pursue other self-treatment options (herbs, etc.)
- Go to pharmacy
- Go to traditional healer

19. What worries you the most when you think about intestinal parasites?

**Awareness and attitude of School-based deworming**

20. Have you heard about school-based deworming for intestinal parasites and bilharzia in your region? Probe about

- Has it been conducted
- When it was conducted
- Where it was conducted
- Length of deworming activity
- Mode of drug distribution and
- Interaction and assistance given to the CHEW.

21. How do you and your fellow ECD teachers feel about the School-based deworming activity? Probe for

- Importance of the school-based deworming activity
- The length of the school-based deworming activity

22. How did you and people in your community learn about the school-based deworming activity?Probe for

- Sources of information
- Sufficiency in informing the communities about the school-based deworming activity
- frequency of informing the communities about the school-based deworming activity
- effectiveness of the source of information

23. Did your ECD child/children take the drugs during the school-based deworming activity?

Probe for:

- Problems if any encountered while assisting the CHEW
- Attendance of children on treatment day
- Time spent while waiting for the CHEW to arrive at the ECD
- Preferred method to use in administering treatment to ECD children next time offered

24. Did your ECD child/children experience any problems after taking the drugs for worms and bilharzia?

Probe for:

- Kind of problems
- How resolved
- Possibility of taking drugs next time offered

25. What is your opinion regarding the school-based deworming activity for intestinal worms and bilharzia?

Probe for

Importance/need

Effectiveness

**THANK YOU VERY MUCH FOR YOUR COOPERATION**

| **In-depth Interview with CHEWs regarding their opinion on reaching the ECD age children during the National School-based deworming**  ID_______________________________________  Time_____________________________________  Date______________________________________  District _____________________________________  Division_____________________________________  Educational Zone **_______________________________**   \| Instructions:  This form should be used for in-depth interviews with the CHEWs.  If the participants refuse to answer a question, circle the number of the question and do not mark any answers for that question.  After obtaining informed consent, read the following instructions to the participants: \| \| --- \| \| **“I am going to ask you questions about the National school based deworming program, so as to collect information about your knowledge of Bilharzia and soil transmitted helminthes and opinion of the National School-based Deworming Programme, your community members preferences for being reaching their ECD Children during the campaign, barriers affecting the programme and existing opportunities and outlets that could be used to reach the ECD children for treatment. Please answer the questions as honestly as you can. Your information which I will tape record will be kept private and this form will not have your name anywhere. All the information will be kept confidential until the conclusion of the study when it will be destroyed. If you have any questions or do not understand what I am asking you at any time, please ask for clarification. Some questions may prove embarrassing to you.**  **Please remember that you do not have to answer any questions that you do not want to answer and you may discontinue the discussion at any time. Do you have any questions before we begin?”** \|   **Socio-Demographic Characteristics**  1. Sex (Tick)  Male (1)  Female (2)  2. Year of birth _____________________.  3. Age in Years _____________________  4. Marital Status (Tick)  Single (1)  Currently Married (2)  Type of Marriage Polygamous (1) Monogamous ( 2 )  Divorced (3)  Widow/ widower (4)  Others (Specify)___________________________  5. Religion (Tick)  Christian (1)  Islam (2)  Non-practicing (3)  Others, specify (4)__________________________  **Actual Interview (Observe - record non-verbal communication)**  **Awareness Creation**   1. How did you get information that you would administer drugs to the ECD children during the National School-based Deworming exercise? 2. How were you selected to administer drugs during the National School-based Deworming exercise? 3. Are there any special reasons as to why you agreed to distribute drugs to the ECD children?   **Training**   1. Could you tell me about the training given for the drug administration? Probe for 2. Who trained you? 3. The content of training 4. Duration of training 5. Venue of training   **Collection of drugs**   1. Did you experience any problems in getting the drugs? Probe for 2. Time spent while waiting to receive the drugs 3. Quantities of drugs received compared to target population   **Mode of drug administration**   1. How did you go about the drug administration? Probe for 2. Presence of the ECD children in school during the treatment day 3. Management of the children at the treatment area 4. Availability of safe water for swallowing drugs |  |  |
| --- | --- | --- | --- | --- |
| 13. Were you comfortable with the way you went about administering the drugs to the ECD Center children? Probe for   1. Assistance received from the ECD teachers 2. Your arrival as the CHEW at the ECD Center on schedule 3. Ability to treat all ECD Center Children registered at each ECD Center as scheduled   14. Do you think you reached all targeted ECD agechildren who are enrolled in the nursery schools assigned to you? Probe for   1. Names and total number of ECD Centers in the area allocated to the CHEW 2. Total number of ECDs that you were able to reach for treatment 3. Possible reasons as to why you were unable to reach some if you did not reach all   15. Did you face any difficulty during drug administration to ECD Center children? Probe for   1. The ECD children’s compliance with treatment 2. Time spent on average while handling one ECD Center 3. Would you recommend that drug administration for the ECD Center children be done the same way in the next round of school-based deworming? Probe for 4. Any facilitating/motivating factors 5. Any barriers/hindering factors 6. Would you be willing to take part in the drug administration to the ECD children in the next round of school-based deworming? Probe for 7. Reasons why willing to take part in the next round or reasons why not willing   **Management of side effects**   1. Could you tell me if there were any problems reported by ECD children after taking the drugs? Probe for  \| No. \| state the problems \| How problem got managed \| Who managed the problem \| \| --- \| --- \| --- \| --- \| \|  \|  \|  \|  \| \|  \|  \|  \|  \| \|  \|  \|  \|  \| \|  \|  \|  \|  \| \|  \|  \|  \|  \| \|  \|  \|  \|  \| \|  \|  \|  \|  \| \|  \|  \|  \|  \| \|  \|  \|  \|  \|   **Record maintenance**  19. Could you tell me about maintenance of records of the drug administration to ECD children? Probe for   - - 1. Availability of enough forms to do the recording of ECD children treatment     2. Ease of recording treatment of ECD children on the forms     3. Availability of ECD children’s bio-data   20a. Can you show us some of these records? Check if   1. Types of documents used are correct 2. Records taken are well done   21. Did you face any problems in making these records? If yes, what problems?  Yes (1)  No (2)  If yes, state the problems  How did you resolve the problems?  If no, give reasons?  **Information, Education and Communication (IEC)**  22. Were the community members in your area of operation informed about school-based deworming and the venue where their ECD age children would receive the treatment?  Yes (1)  No (2)  23. State how the community members got the information about the school-based deworming activity and the venue for treating the ECD age children?  24. Do you think that the methods through which the community got information on the school-based deworming activity was adequate?  Yes (1)  No (2)  If yes, state the reasons  If no, give reasons?  If no, what suggestions can you give for improving way of giving information?  25a. Were you involved in informing the community about the school-based deworming activity?  Yes (1)  No (2)  If yes, state the approaches you used  If no, give reasons?  **Incentives**  26. Did you receive any support in your role of administering treatment? (Tick)  Yes (1)  No (2)  If yes, state the kind of support (Financial, moral etc.)  If yes, whom did you receive the support from? (in decreasing order)  If no, give reasons?  27. Who would you say has been most supportive?  28. What did they do in particular?  29. Who would you say has been least supportive?  30. What do you think they had in their powers to do but failed to do so in your support?  31. Was the scheduled time for drug administration satisfactory? (Tick)  Yes (1)  No (2)  If yes, state the reasons  If no, give reasons?  If no, what length of time do you think is satisfactory??  **THANK YOU VERY MUCH FOR YOUR COOPERATION** | | |

**In-depth interview for Primary School Teachers administering treatment to ECD age children.**

Date: _____________________________________

Participant ID: ________________________________________

Age: _____________________________________

District: _______________________________________

Division: ______________________________________

Educational Zone: _______________________________________

School Name: ____________________________________________

| Instructions:  This form should be used for interviews with Primary School Teachers.  After obtaining informed consent, read the following instructions to the participant:  If the participant refuses to answer a question, circle the number of the question and do not mark any answers for that question. |
| --- |
| “ **I am going to ask you questions about the National school based deworming program, whether the children in the ECD schools feeding into your school were treated for STH and Bilharzia, your willingness to continue treating ECD aged children at your primary school and your preference for reaching these children during subsequent deworming activities. Please answer the questions as honestly as possible. Your information will be kept private and this form will not have your names anywhere, you will be identified by a number only. If you have any questions or do not understand what I am asking you at any time, please ask for clarification. Some questions may prove embarrassing to you.**  **Please remember that you do not have to answer any questions that you do not want to answer and you may discontinue the interview at any time. Do you have any questions before we begin?”** |

**Socio-Demographic Characteristics**

1. Sex (Tick)

Male (1)

Female (2)

2. Year of birth _____________________.

3. Age in Years _____________________

4. Marital Status (Tick)

Single (1)

Currently Married (2) Type of Marriage Polygamous (1) Monogamous (2)

Divorced (3)

Widow/ widower (4)

Others (Specify)___________________________

5. Religion (Tick)

Christian (1)

Islam (2)

Non-practicing (3)

Others, specify (4)__________________________

**Ice breaker**

Let us now begin. Let’s find out some more about who we are. You will tell us your name and introduce yourself very briefly, for example where you live and anything else you would like to mention about yourself.

**[→Start recorder after this has been done]**

**Awareness Creation**

1. How did you get information that you would administer drugs during the National School-based Deworming exercise?
2. How were you selected to administer drugs during the National School-based Deworming exercise?
3. Are there any special reasons as to why you agreed to administer the drugs?

**Training**

1. Could you tell me about the training given for the drug administration? Probe for
2. Who trained you?
3. The content of training
4. Duration of training
5. Venue of training

**Collection of drugs**

1. Did you experience any problems in getting the drugs? Probe for
2. Receiving the drugs on time
3. Receiving sufficient quantities of drugs

**Mode of drug administration**

1. How did you go about the drug administration? Probe for
2. Arrangement at the treatment area
3. Management of the children at the treatment area
4. Ability to treat the ECD Center children first i.e. as they arrive

7. Were you comfortable with the way you went about administering the drugs to the ECD Center children? Probe for

1. Assistance received from the ECD teachers
2. Arrival of ECD Center children in the morning
3. Ability to treat all ECD Center Children without keeping them waiting for a long time

8. Do you think you reached all targeted ECD age children who are enrolled in your nearby nursery schools? Probe for

1. Names and total number of ECDs Centers near your primary school
2. Total number of ECDs Centers that came to the primary school for treatment
3. Possible reasons as to why those that did not come failed to come

9. Did you face any difficulty during drug administration to ECD Center children? Probe for

1. The ECD children’s compliance with treatment
2. Time spent on average while treating an ECD group
3. Management of the ECD children

10. Would you recommend that drug administration for the ECD Center children be done the same way in the next round of school-based deworming?

Yes (1)

No (2)

If yes, give reasons

If no, give reasons?

If no, give suggestions how you would want it done next time.

1. Would you be willing to take part in the drug administration to the ECD children in the next round of school-based deworming?

Yes (1)

No (2)

If yes, give reasons (what is the motivating factor)

If no, give reasons

**Management of side effects**

1. Did any ECD children report any problems after taking the drugs?

Yes (1)

No (2)

If yes, state the problems

| No. | state the problems | How problem got managed | Who managed the problem |
| --- | --- | --- | --- |
|  |  |  |  |
|  |  |  |  |
|  |  |  |  |
|  |  |  |  |
|  |  |  |  |
|  |  |  |  |
|  |  |  |  |
|  |  |  |  |
|  |  |  |  |

**Record maintenance**

13. Could you tell me about maintenance of records of the drug administration to ECD children? Probe for

1. Availability of enough forms to do the recording of ECD children treatment
2. Ease of recording treatment of ECD children on the forms
3. Availability of ECD children’s bio-data

13a. Can you show us some of these records? Check if

1. Types of documents used are correct
2. Records taken are well done

14. Did you face any problems in making these records?

Yes (1)

No (2)

If yes, state the problems

How did you resolve the problems?

If no, give reasons?

14a. How did you resolve them?

**Information, Education and Communication (IEC)**

15. Were the community members around your school informed about the school-based deworming activity and where to take their ECD age children?

Yes (1)

No (2)

16. State how the community members got the information about the school-based deworming activity and the venue for treating the ECD age children?

17. Do you think that the methods through which the community got information on the school-based deworming activity was adequate?

Yes (1)

No (2)

If yes, state the reasons

If no, give reasons?

If no, what suggestions can you give for improving the way of giving information?

18. Were you involved in informing the community about the school-based deworming activity?

Yes (1)

No (2)

If yes, state the approaches you used

If no, give reasons?

**Incentives**

19. Did you receive any support in your role of administering treatment? (Tick)

Yes (1)

No (2)

If yes, state the kind of support (Financial, moral etc)

If yes, whom did you receive the support from? (in decreasing order)

If no, give reasons?

20. Who would you say has been most supportive?

21. What did they do in particular?

22. Who would you say has been least supportive?

23. What do you think they had in their powers to do but failed to do so in your support?

24. Was the scheduled time for drug administration satisfactory? (Tick)

Yes (1)

No (2)

If yes, state the reasons

If no, give reasons?

If no, what length of time do you think is satisfactory??

**THANK YOU VERY MUCH FOR YOUR COOPERATION**

**In-depth Interviews with Opinions Leaders regarding their perception of treatment of ECD age children for bilharzia and STHs during the National School-Based Deworming Programme**

ID_______________________________________

Time_____________________________________

Date______________________________________

District _____________________________________

Division_____________________________________

Educational Zone **_______________________________**

| Instructions:   - This form should be used for in-depth interviews with the Opinion Leaders. - If the participants refuse to answer a question, circle the number of the question and do not mark any answers for that question. - After obtaining informed consent, read the following instructions to the participants: |
| --- |
| **“I am going to ask you questions about the National school based deworming program, so as to collect information about your knowledge of Bilharzia and soil transmitted helminthes and opinion of the National School-based Deworming Programme, your community members preferences for reaching their ECD Children during the campaign, barriers affecting the programme and existing opportunities and outlets that could be used to reach the ECD children for treatment. Please answer the questions as honestly as you can. Your information which I will tape record will be kept private and this form will not have your name anywhere. All the information will be kept confidential until the conclusion of the study when it will be destroyed. If you have any questions or do not understand what I am asking you at any time, please ask for clarification. Some questions may prove embarrassing to you.**  **Please remember that you do not have to answer any questions that you do not want to answer and you may discontinue the discussion at any time. Do you have any questions before we begin?”** |

**Socio-Demographic Characteristics**

1. Sex (Tick)

Male (1)

Female (2)

2. Year of birth _____________________.

3. Age in Years _____________________

4. Marital Status (Tick)

Single (1)

Currently Married (2) Type of Marriage Polygamous (1) Monogamous (2)

Divorced (5)

Widow/ widower (6)

Others (Specify)

4. Religion (Tick)

Christian (1)

Islam (2)

Non-practicing (3)

Others, specify (4)__________________________

**Socio-economic characteristics**

5. Level of Education (Tick)

Never attended school (1)

Did not complete primary school (2)

Completed primary school but did not complete secondary school (3)

Completed secondary school (4)

Further studies after secondary school (5)

Others, specify (6)___________________________

6. Main occupation (Tick)

Farmer (1)

Small business (kiosk, kibanda) (2)

Big business (shop) (3)

Housewife (4)

Salaried worker (teacher, police, chief) (5)

Fisherman (6)

Casual laborer (7)

Others, specify (8)________________________

**Actual Interview (Observer - record non-verbal communication)**

| **Questions** |  |
| --- | --- |
| 1. Could you tell me about the National School-based Deworming Programme for Bilharzia and soil transmitted helminthes control in your community? Probe for |  |
| 1. Who did it? |  |
| 1. When was it done? |  |
| 1. How was it done? |  |
| 1. Why was it done? |  |
| 2. How did people in your community know about the School-Based Deworming Programme? Probe for |  |
| 1. Adequacy of information |  |
| 1. Enough time given to understand the information |  |
| 1. Too much or too little information given |  |
| 1. Period between that which the information is given and the drugs are distributed is too soon or too long |  |
| 3. How was the participation of the ECD age children in the treatment for bilharzia and STHs control? Probe for |  |
| 1. Any problems or barriers |  |
| 1. Any facilitation given |  |
| 4. What would you say about?   1. Treating of ECD children at their ECD Centres |  |
| 1. The use of the Health workers to treat the ECD children |  |
| 5. How can drug administration among the ECD age children be improved? Probe about   1. Administering drugs at ECD Centres 2. Use of Health workers to treat the ECD children 3. Duration of distribution 4. Awareness creation methods |  |

**THANK YOU VERY MUCH FOR YOUR COOPERATION**
